# Are your covariates under control? How normalization can re-introduce covariate effects

**DOI:** 10.1038/s41431-018-0159-6

**Published:** 2018-04-30

**Authors:** Oliver Pain, Frank Dudbridge, Angelica Ronald

**Affiliations:** 1 Department of Psychological Sciences, Birkbeck, University of London, London, UK; 2 Department of Non-communicable Disease Epidemiology, London School of Hygiene and Tropical Medicine, London, UK; 3 Department of Health Sciences, University of Leicester, Leicester, UK

## Abstract

Many statistical tests rely on the assumption that the residuals of a model are normally distributed. Rank-based inverse normal transformation (INT) of the dependent variable is one of the most popular approaches to satisfy the normality assumption. When covariates are included in the analysis, a common approach is to first adjust for the covariates and then normalize the residuals. This study investigated the effect of regressing covariates against the dependent variable and then applying rank-based INT to the residuals. The correlation between the dependent variable and covariates at each stage of processing was assessed. An alternative approach was tested in which rank-based INT was applied to the dependent variable before regressing covariates. Analyses based on both simulated and real data examples demonstrated that applying rank-based INT to the dependent variable residuals after regressing out covariates re-introduces a linear correlation between the dependent variable and covariates, increasing type-I errors and reducing power. On the other hand, when rank-based INT was applied prior to controlling for covariate effects, residuals were normally distributed and linearly uncorrelated with covariates. This latter approach is therefore recommended in situations were normality of the dependent variable is required.

## Introduction

Many statistical tests rely on the assumption that the residuals of a model are normally distributed [1]. In genetic analyses of complex traits, the normality of residuals is largely determined by the normality of the dependent variable (phenotype) due to the very small effect size of individual genetic variants [2]. However, many traits do not follow a normal distribution. In behavioral research in particular, questionnaire data often exhibit marked skew as well as a large number of ties between individuals. The non-normality of residuals can lead to heteroskedasticity (comparison of variables with unequal variance) potentially resulting in increased type-I error rates and reduced power [3]. However, most genome-wide analysis software currently implements only linear and logistic models, precluding the use of more general parametric or non-parametric analyses.

There are several approaches to either satisfy the normality assumption or control for violations of it. One of the most popular is the transformation of the dependent variable to follow a normal distribution, i.e., normalization. There are several transformations that can be used for this purpose, the most popular being log, power, or Box-Cox transformations, and rank-based inverse normal transformations (INTs), also referred to as quantile normalizations, such as the Van de Waerden transformation [4]. In many cases the use of log transformation has been shown to be insufficient for normalizing data. Conversely, rank-based INTs always create a perfect normal distribution when there are no tied observations. Previous studies have reported that although rank-based INTs can lead to loss of information, this approach controls power and type-I error rate [5, 6]. However, a comprehensive review of rank-based INTs demonstrated that in certain scenarios, rank-based INTs do not control type-I error, although they remain useful in large samples where alternative methods, such as resampling, are less practical [4]. Furthermore, normalization provides other practical advantages when pooling data from different sources in which the residual distributions may also vary.

It is often desirable to adjust for covariates in analysis. In genetic studies, principal components of ancestry are commonly included to reduce confounding by population structure. When a transformation to normality is used, the covariates may be included in the analysis model after transformation, or alternatively they may be regressed against the response prior to the residuals being transformed to normality. The latter approach has been used in a number of recent high-profile studies [7–9] and is also automated in the “rntransform” function within GenABEL, a popular R package [10]. One reason is that confounders may be considered to have their effects on the untransformed, rather than the normalized, variable. Another reason is that pre-adjustment for covariates will break many of the ties that are present in data derived from questionnaires or other rating scales that are usually represented by a small number of discrete values.

This study investigates the effect of first regressing out covariate effects from quantitative dependent variables, before applying rank-based INTs to the resulting residuals. We use simulations to study the consequences of this procedure, varying the degree of skew in the dependent variable, proportion of tied observations in the dependent variable, and the original correlation between the dependent variable and covariate. We then explore an alternative approach whereby rank-based INT is first applied to the dependent variable (randomly splitting tied observations) before regressing out covariate effects. We demonstrate that regressing covariate effects from the dependent variable creates a covariate-based rank, which is subsequently distorted by rank-based INT, leading to increased type-I errors and reduced power. Our results suggest that the practice of regressing out covariate effects prior to transformation should be discouraged. As an alternative we suggest that when strict normality of the dependent variable is required, rank-based INT should be performed before controlling for covariates, with random ranking of tied observations.

## Materials and methods

### Simulation of phenotypic data

Two types of phenotypic data were simulated: quantitative variables containing no tied observations (herein referred to as continuous variables) and quantitative variables containing tied observations (herein referred to as questionnaire-type variables). These variables were simulated to exhibit different degrees of skew ranging from −2 to 2. Skewed variables were created using the R “rbeta” function, which randomly generates numbers following a beta distribution with two shape parameters to control the degree of skew. Ten thousand observations were simulated for each variable. To create tied observations in the questionnaire-type variables, the initially continuous data were collapsed into evenly distributed and discrete response bins. The number of response bins, determining the proportion of tied observations, was varied between 5 and 160.

The R functions used to create continuous and questionnaire-type variables, called “SimCont” and “SimQuest” respectively, are available in Supplementary Text 1 and 2.

A normal distribution is defined by skew = 0 but also kurtosis = 0. Given that the simulated variables were generated to follow a beta distribution, variables with a skew equal to zero may not have a kurtosis equal to zero. To ensure that the correction of kurtosis was not driving effects seen when skew is equal to zero, continuous and questionnaire-type variables were also generated using the “rnorm” function in R to exhibit both a skew and kurtosis of zero. The functions used to create continuous and questionnaire-type with skew and kurtosis fixed to zero, called “SimContNorm” and “SimQuestNorm” respectively, are available in Supplementary Text 3 and 4.

Skew and kurtosis were measured using the “skewness” and “kurtosis” functions from the R package “e1071” [11].

### Simulation of covariate data

To create correlated covariate data, noise was added to each simulated phenotypic variable until the desired phenotype–covariate correlation was achieved. Phenotype–covariate correlations (Pearson’s) were varied between −0.5 and 0.5. Noise was added to the questionnaire variables using the “jitter” function in R.

The R function used to create covariates for each phenotypic variable, called “CovarCreator”, is available in Supplementary Text 5.

### Normalization after adjusting for covariates

Linear regression of each covariate against the corresponding phenotypic variable was used to calculate phenotypic residuals, which are linearly uncorrelated with the covariates. The Spearman’s rank correlation between the residuals and covariates was measured. The residuals were then normalized using the “rntransform” from the GenABEL package in R, which applies a rank-based INT similar to van de Waerden transformation. To determine whether the transformed residuals were still linearly uncorrelated with covariates, the Pearson correlation between the transformed residuals and covariates was calculated.

### Normalization before adjusting for covariates

This was carried out using the same simulated questionnaire-type and continuous variables and covariates. The raw questionnaire-type and continuous variables underwent rank-based INT using a modified version of the “rntransform” function from GENABEL that randomly ranks any tied observations. The modified version of “rntransform”, called “rntransform_random”, is available in Supplementary Text 6. Linear regression of each covariate against the corresponding normalized variables was used to calculate phenotypic residuals, which are linearly uncorrelated with the covariates.

One concern with rank-based INT, particularly when randomly splitting ties, is that the linear relationship between the phenotypic variable and independent variables (including covariates) may be severely distorted. To determine the extent to which rank-based INT when randomly splitting ties distorts phenotypic variables, the Pearson correlations between the untransformed and transformed phenotypic variables were calculated. To determine the extent to which rank-based INT when randomly splitting ties distorts the relationship between the phenotypic variables and covariates, the Pearson correlation between the transformed phenotypic variables and covariates was calculated.

Another concern with normalizing the phenotypic variable before regressing out covariates is that the process of regressing out covariates may re-introduce skew in the residuals. We therefore calculated the skew of the residuals after regressing out covariates.

### Demonstration using real data

To determine whether the predicted effects (when using simulated data) of performing rank-based INT before or after regressing out covariate effects are seen in practice, the same procedures were applied to real questionnaire data provided by the Twins Early Development Study (TEDS) [12]. Data from two questionnaires were used measuring Paranoia and Anhedonia. Both of these measures are part of the SPEQ (Specific Psychotic Experiences Questionnaire) [13]. Individuals with missing phenotypic data were excluded from all analyses. Sum scores of unrelated individuals were calculated by summing the response of each item. Each item of both the Paranoia and Anhedonia scales were coded as values from 0–5, with the total ranges of the Paranoia and Anhedonia scales being 0–75 and 0–50, respectively. Sum scores were calculated using different numbers of items (1, 2, 4, 8) to create different numbers of response bins (5, 10, 20, 40) and as in the simulation study. The covariates used were age (continuous variable skew of −0.32) and sex (binary variable with skew of 0.22). Table 1 shows the skew, number of response bins (proportion of ties), and correlation with covariates for each of dependent variable. The TEDS data were analyzed using the same procedure as the simulated data.

**Table 1:** Skew, range, and correlation with covariates for dependent variables derived from TEDS sample

Dependent variable	Range	Skew	Pearson correlation with age	Pearson correlation with sex
Paranoia	5	1.357	0.055	0.018
Paranoia	10	1.195	0.043	−0.026
Paranoia	20	1.095	0.03	−0.022
Paranoia	40	1.296	0.022	−0.059
Anhedonia	5	1.868	−0.006	0.177
Anhedonia	10	0.858	−0.025	0.127
Anhedonia	20	0.651	−0.02	0.135
Anhedonia	40	0.537	−0.013	0.205

## Results

### Normalization after adjusting for covariates

As expected, regressing covariates against phenotypic variables created phenotypic residuals that were linearly uncorrelated with covariates. Although there was no linear correlation, in almost all simulations a rank-based correlation remained between the residuals and covariates (Supplementary Figure 1–7). As a consequence, rank-based INT of residuals re-introduced a linear correlation between the phenotypic variables and covariates (Supplementary Figure 8–14). Three factors predicted to affect the re-introduction of correlation between the phenotypic variables and covariates were tested. These factors were the original skew of the phenotypic variable, the original correlation between the phenotypic variable and covariate, and the proportion of tied observations in the original phenotypic data.

**Fig. 1:**
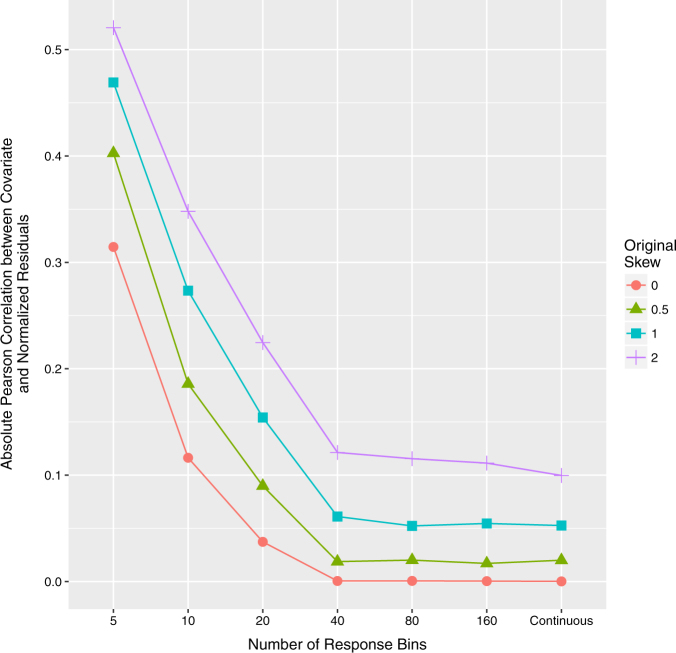
The relationship between the number of available responses (*x*-axis) and correlation between normalized residuals and covariate (*y*-axis) for different values of the skew in the raw phenotypic data. Within this figure, the correlation between the untransformed phenotypic data and covariate data is at 0.06

First, in terms of skew, greater skew of the phenotypic variable was associated with a higher correlation between the normalized phenotypic residuals and the covariate data (Fig. 1, Supplementary Figure 8–14). The direction of skew had no effect on the correlation between the normalized residuals and the covariate data. The effect of normalizing residuals when skew was equal to zero remained when kurtosis was also fixed to zero (Supplementary Figure 15).

Second, the direction of the original correlation between the original phenotypic variable and the covariates was reversed after rank-based INT of residuals. The magnitude of correlation between the original dependent variable and covariates showed different effects according to the proportion of ties. In questionnaire-type data, when the proportion of tied observations was high, the magnitude of correlation between the original questionnaire data and covariates had a negative relationship with the degree to which normalization re-introduced the correlation with covariates (Supplementary Figure 8). However, this negative relationship reversed as the proportion of ties decreased (Supplementary Figure 16). This means that when the proportion of tied observations was low (or in continuous data), the magnitude of correlation between the original questionnaire data and covariates had a positive relationship with the degree to which normalization re-introduced the correlation with covariates.

Third, independent of the original correlation between the dependent variable and covariates, the proportion of ties in the phenotypic variable influenced the extent to which normalization of residuals reintroduced a correlation with covariates. A decreased number of response bins in the questionnaire-type data (i.e., smaller range and more tied observations) resulted in an increased correlation between covariates and normalized residuals (Fig. 1). However, even when there were 160 response bins, or the data were continuous, rank-based INT still re-introduced a correlation with covariates when the data had an original skew >0.5 (Supplementary Figure 13 and 14).

As previously mentioned, although there is no linear correlation between phenotypic residuals and covariates, a rank-based correlation between the phenotypic residuals and covariates remained in almost all simulations. The factors affecting the magnitude of rank-based correlation between phenotypic residuals and covariates are the same as those influencing the effect of rank-based INT of residuals (Supplementary Figure 17–18).

### Normalization before adjusting for covariates

Rank-based INT of phenotypic variables, randomly splitting ties, before subsequent regression of covariates against the normalized phenotypic data, always resulted in phenotypic residuals with no linear correlation with covariates, and in the majority of simulations, skew less than 0.05.

The correlations between the phenotypic variables and the covariates decreased a small amount (median 5%) after rank-based INT of the phenotypic variables, compared to their original correlations (Supplementary Table 1). The extent to which the correlation decreased was dependent on the original correlation, the skew of the dependent variable, and the proportion of tied responses in the dependent variable (Supplementary Figures 19–24).

Comparing the original values of the dependent variables to their values after rank-based INT (randomly splitting tied observations) yielded correlations between 0.77 and 1.00. An increased proportion of tied observations and increased skew led to a decreased correlation after rank-based INT (Supplementary Figure 25).

Regressing covariates after normalizing the dependent variables introduced a smaller degree of skew, when covariates had either a low skew themselves or a low correlation with the dependent variable. The degree to which regressing covariate effects introduced skew was not dependent on the proportion of tied observations. Overall, regressing covariates introduced a small amount of skew to the dependent variable (0.00–0.11) unless the covariate had a correlation with the dependent variable over 0.25 and a skew greater than 0.05 (Supplementary Figure 26). However, highly skewed covariates may introduce larger amounts of skew even when exhibiting a low correlation with the dependent variable.

### Real data analysis

The effect of applying INTs to residuals in simulated questionnaire-type data was then observed in real questionnaire data from TEDS. When using the age covariate (continuous) the magnitude and direction of effect of applying INT to residuals were similar to those of simulated questionnaire-type data (Supplementary Table 2–3). The effect of INT on residuals when using real questionnaire data was slightly reduced in comparison to effects observed, when using simulated questionnaire-type data.

When the sex covariate (binary) was used, the magnitude, and in some cases the direction, of the effect of rank-based procedures varied from effects observed in simulated data. Although regressing the effect of a binary covariate altered the outcome of rank-based procedures, application of rank-based procedures to residuals still re-introduced a correlation with covariates (Supplementary Table 4–5). Importantly, when a dichotomous variable was used, a large number of ties in the data still existed reducing the efficacy of rank-based INT.

We then applied rank-based INT (randomly splitting tied observations) before regressing out covariate effects. The results in real questionnaire data were comparable to the effects observed when using simulated data. Rank-based INT, randomly splitting ties, and subsequent regression of covariates created residuals that were linearly uncorrelated with covariates and normally distributed (Supplementary Table 6–7). The correlation between the dependent variable and covariate did vary slightly before and after rank-based INT (Supplementary Table 6–7). Contrary to the observed effects when using simulated data, the correlation between the dependent variable and the covariate did not always decrease. The Pearson correlation between raw and normalized questionnaire data varied between 0.83 and 0.99 dependent on the skew of the raw data and the number of response bins (Supplementary Table 8). Similar to the results of our simulations, the effect of regressing covariates out of the normalized variables did not re-introduce skew greater than 0.02 in any situation (Supplementary Table 6–7).

## Discussion

**Fig. 2:**
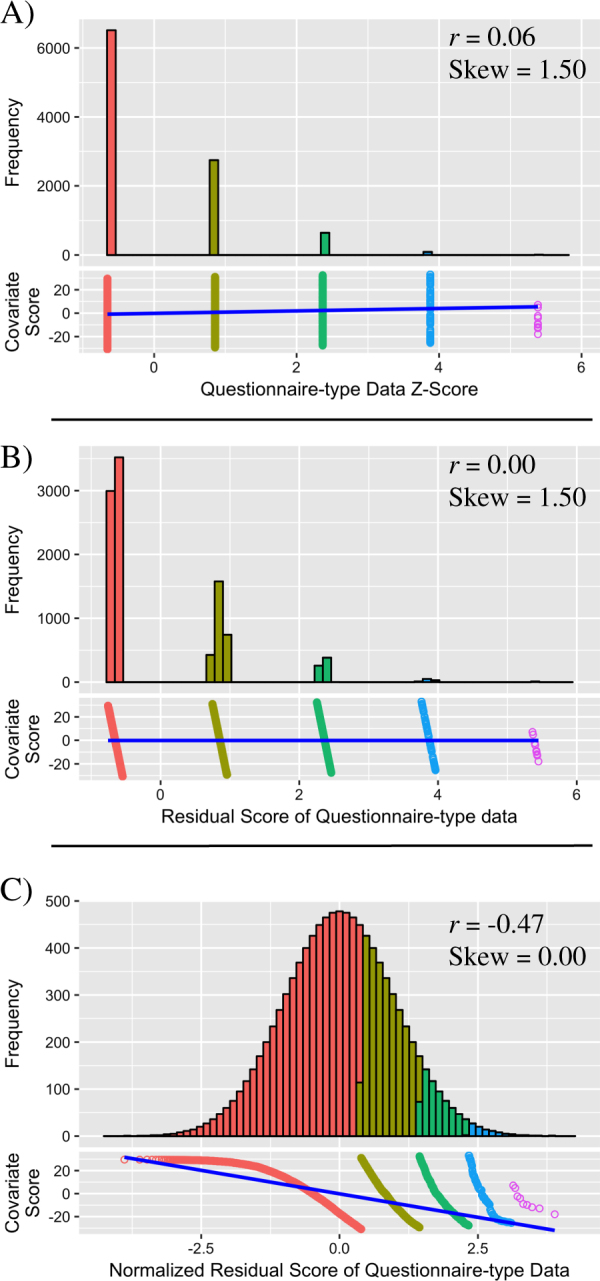
The effect of applying a rank-based INT to residuals of questionnaire-type data, i.e., after regressing out covariates. All correlations referred to in this figure are Pearson (linear) correlations. **a** Untransformed questionnaire-type variable and its relationship with a continuous covariate. The questionnaire-type variable has a range of 5. A weak linear relationship exists between the questionnaire-type variable and covariate. **b** Questionnaire-type variable residuals after regressing out the relationship with the covariate. No linear relationship exists between the questionnaire-type residuals and covariate. Regressing out covariate effects has led to the separation of many tied observations, creating a covariate-based rank within the questionnaire-type variable residuals. **c** After the rank-based INT of questionnaire-type variable residuals, the transformed questionnaire-type variable residuals show a strong linear correlation with the covariate. This correlation is stronger and in the opposite direction to the original correlation between the untransformed questionnaire-type variable and the covariate

This study has demonstrated that regressing covariates against the dependent (phenotypic) variable and then using rank-based INT to transform the residuals to normality re-introduces a correlation between the covariates and the normalized dependent variable. This effect occurs because the process of regressing covariates against the response variable leads to a covariate-based rank in the residuals, which is then used to redistribute the data (Fig. 2). This effect of regressing covariates against response variables occurs when the response variable is continuous (contains no tied observations) or questionnaire-type (contains tied observations), however the effect increases as the proportion of tied observations increases. The degree to which the covariate correlation is re-introduced during rank-based INT is dependent on the original skew of the response variable, although when the data contain a large proportion of tied observations, a correlation with covariates is re-introduced even when there is no skew.

**Fig. 3:**
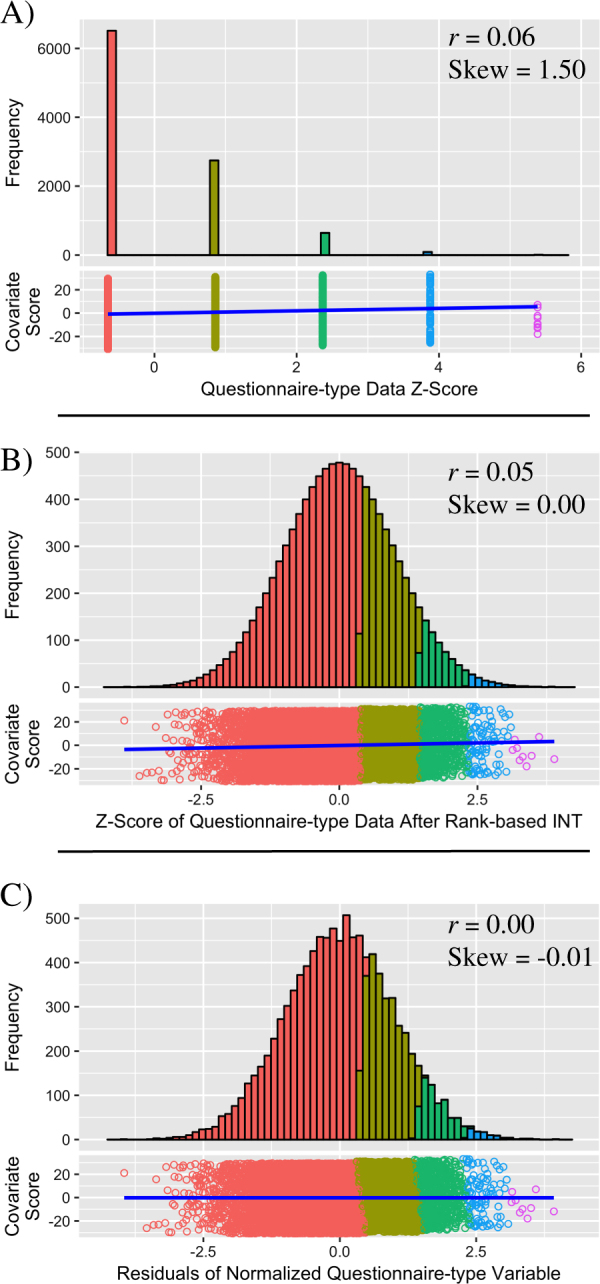
The effect of applying a rank-based INT to questionnaire-type data before regressing out covariates. All correlations referred to in this figure are Pearson (linear) correlations. **a** Untransformed questionnaire-type variable and its relationship with a continuous covariate. The questionnaire-type variable has a range of 5. A weak linear relationship exists between the questionnaire-type variable and covariate. **b** Questionnaire-type variable after rank-based INT, randomly splitting tied observations. Relationship between the questionnaire-type variable remains intact. **c** Covariate effects have been regressed from the normalized questionnaire-type variable. There is no linear relationship between the residuals and the covariate, and the skew is close to zero

This study has also evaluated an alternative procedure for preparing data for parametric analyses, whereby the response variable undergoes rank-based INT, randomly separating ties, before regressing out covariate effects. Our findings demonstrate that this alternative approach is preferable as it creates a normally distributed response variable with no correlation with covariates (Fig. 3). The notion of normalizing the response variables before estimating its relationship with covariates may seem counterintuitive as the process of normalization may disrupt the true relationship between variables. Although this may be true in some scenarios, when the variables are skewed and/or contain tied observations, the change in relationship between variables due to normalization (Supplementary Table 6–7) is small relative to the change in relationship when normalizing residuals (Supplementary Table 2 and 4). In contrast, regressing covariates after normalization will leave no correlation between the variables, meaning that any confounding by those covariates will be eliminated. A limitation of applying rank-based INT before controlling for covariates is the requirement of randomly splitting ties. This process will introduce random variation in the data, subsequently reducing statistical power. However, the alternative approach of normalizing after controlling for covariates introduces non-random variation, leading to a reduction in power and confounding.

Given the importance of phenotypic transformations, authors must describe the details of this process. Many studies do not clearly describe the details in which the data are processed, but there are some major studies that have clearly applied rank-based INT to residuals [7–9]. We do not believe that the results of these studies are seriously in error as they have either dealt with traits that have a very low skew and/or are continuous, or they have replicated their findings using binary outcomes based on untransformed data. However, we do believe that the potential problems with rank-based INT of residuals are not well known, and that researchers should be aware of these issues before applying such a procedure. There are, of course, many parametric and non-parametric methods that do not require normality in residuals, and in an ideal world one would identify and apply a model that accurately describes the data at hand. However, this is not always practical in large genetic studies with many contributing datasets, and rank-based INTs remain a pragmatic approach of choice in spite of its well-known limitations [4]. We suggest that, if rank-based INTs must be used, researchers should adjust for covariates after rather than before applying the normalizing transformation.

These findings are not just relevant to rank-based INT of residuals but highlight the importance of procedures that introduce or alter the rank of observations. Another procedure that will alter the rank-based relationship between variables is the calculation of factor scores via principal components analysis (PCA). PCA is a method that applies orthogonal transformation to identify linearly uncorrelated axes of variation among observations. Although the derived factors are linearly uncorrelated, they may have a rank-based correlation. Therefore, if the factors are skewed, subsequent rank-based INT will introduce a linear correlation between factors. Similar to the example of normalizing residuals, if the original correlation between the latent variables is positive, rank-based INT will lead to a negative correlation between derived factors.

Although we conclude that normalization of the dependent variable should be performed prior to adjusting for covariates, regressing out covariates that are either highly skewed or highly correlated with the dependent variable may introduce substantial skew to the residuals. However, this scenario may be unlikely.

In conclusion, this study has demonstrated that rank-based INT of phenotypic residuals after adjusting for covariates can lead to an overcorrection of covariate effects leading to a correlation in the opposite direction between the normalized phenotypic residuals and covariates, and in questionnaire-type data, often of a greater magnitude. This finding has implications for all rank-based procedures and highlights the importance of clearly documenting how the raw data are handled. Normalization of phenotypic data before regressing out covariates has been shown to produce normally distributed phenotypic residuals that are uncorrelated with covariates, and is therefore recommended in situations when rank-based INT is the pragmatic choice.

## Electronic supplementary material

Supplementary Material
