# Supplementary material for: Are your covariates under control? How normalization can re-introduce covariate effects
Source: Eur J Hum Genet. 2018 Apr 30;26(8):1194–201. doi: 10.1038/s41431-018-0159-6 (PMC6057994; doi:10.1038/s41431-018-0159-6)
Supplement: Supplementary file 1 — Supplementary Material [file 41431_2018_159_MOESM1_ESM.docx]

**Supplementary material for ‘Are Your Covariates Under Control? How Normalization Can Re-introduce Covariate Effects’ by Pain *et al*.**

***Supplementary Texts:***

**Supplementary Text 1:** ‘SimCont’ – Function to simulate continuous variables.

**Supplementary Text 2:** ‘SimQuest’ – Function to simulate questionnaire-type variables.

**Supplementary Text 3:** ‘SimContNorm’ – Function to simulate continuous variables with skew and kurtosis equal to zero.

**Supplementary Text 4:** ‘SimQuestNorm’ – Function to simulate questionnaire-type variables with skew and kurtosis equal to zero.

**Supplementary Text 5:** ‘CovarCreator’ – Function to create correlated covariates for continuous and questionnaire-type variables.

**Supplementary Text 6:** ‘rntransform_random’ – Function to perform rank-based INT whilst randomly splitting tied observations.

***Supplementary Tables:***

**Supplementary Table 1:** Difference in covariate correlation with the dependent variable before and after rank-based INT when splitting tied observations randomly.

**Supplementary Table 2:** Effect of rank-based INT of real questionnaire data after regressing out the effect of age.

**Supplementary Table 3:** Effect of regressing out the effect of age from real questionnaire data on Spearman correlation.

**Supplementary Table 4:** Effect of rank-based INT of real questionnaire data after regressing out the effect of sex.

**Supplementary Table 5:** Effect of regressing out the effect of sex from real questionnaire data on Spearman correlation.

**Supplementary Table 6:** Effect of rank-based INT (randomly ranking tied observations) on the relationship between real questionnaire variable and continuous covariate (age).

**Supplementary Table 7:** Effect of rank-based INT (randomly ranking tied observations) on the relationship between real questionnaire variable and binary covariate (sex).

**Supplementary Table 8:** Effect of rank-based INT of real questionnaire variable when randomly ranking tied observations.

***Supplementary Figures:***

**Supplementary figure 1:** Rank-based correlation between simulated questionnaire-type variables (with range of 5) and covariates after regressing out covariate effects.

**Supplementary figure 2:** Rank-based correlation between simulated questionnaire-type variables (with range of 10) and covariates after regressing out covariate effects.

**Supplementary figure 3:** Rank-based correlation between simulated questionnaire-type variables (with range of 20) and covariates after regressing out covariate effects.

**Supplementary figure 4:** Rank-based correlation between simulated questionnaire-type variables (with range of 40) and covariates after regressing out covariate effects.

**Supplementary figure 5:** Rank-based correlation between simulated questionnaire-type variables (with range of 80) and covariates after regressing out covariate effects.

**Supplementary figure 6:** Rank-based correlation between simulated questionnaire-type variables (with range of 160) and covariates after regressing out covariate effects.

**Supplementary figure 7:** Rank-based correlation between simulated continuous variables and covariates after regressing out covariate effects.

**Supplementary figure 8:** Effect of rank-based INT after regressing out covariate effects in simulated questionnaire-type data with range of 5.

**Supplementary figure 9:** Effect of rank-based INT after regressing out covariate effects in simulated questionnaire-type data with range of 10.

**Supplementary figure 10:** Effect of rank-based INT after regressing out covariate effects in simulated questionnaire-type data with range of 20.

**Supplementary figure 11:** Effect of rank-based INT after regressing out covariate effects in simulated questionnaire-type data with range of 40.

**Supplementary figure 12:** Effect of rank-based INT after regressing out covariate effects in simulated questionnaire-type data with range of 80.

**Supplementary figure 13:** Effect of rank-based INT after regressing out covariate effects in simulated questionnaire-type data with range of 160.

**Supplementary figure 14:** Effect of rank-based INT after regressing covariates in simulated continuous data.

**Supplementary figure 15:** Effect of rank-based INT after regressing covariates when kurtosis and skew are equal to zero.

**Supplementary figure 16:** Relationship between proportion of ties and magnitude of original covariate correlation on correlation with covariates after rank-based INT of residuals.

**Supplementary figure 17:** Effect of number of response bins on the rank-based correlation with covariates after regressing out covariate affects.

**Supplementary figure 18:** Relationship between proportion of ties and magnitude of original covariate correlation on rank-based correlation with covariates after regressing out covariate effects.

**Supplementary figure 19:** Difference in covariate correlation with dependent variable after rank-based INT. Original covariate correlation in this figure was 0.5.

**Supplementary figure 20:** Difference in covariate correlation with dependent variable after rank-based INT. Original covariate correlation in this figure was 0.25.

**Supplementary figure 21:** Difference in covariate correlation with dependent variable after rank-based INT. Original covariate correlation in this figure was 0.12.

**Supplementary figure 22:** Difference in covariate correlation with dependent variable after rank-based INT. Original covariate correlation in this figure was 0.06.

**Supplementary figure 23:** Difference in covariate correlation with dependent variable after rank-based INT. Original covariate correlation in this figure was 0.03.

**Supplementary figure 24:** Difference in covariate correlation with dependent variable after rank-based INT. Original covariate correlation in this figure was 0.01.

**Supplementary figure 25:** Correlation between the dependent variable before and after rank-based INT (randomly splitting tied observations).

**Supplementary figure 26:** Magnitude of skew reintroduced when regressing out covariate effects from normalized dependent variables. Proportion of ties did not affect this. This figure is based on simulated continuous variables.

**Supplementary Text 1:** ‘SimCont’ – Function to simulate continuous variables.

SimCont<-function(DesiredSkew, Seed=10101, NumOfSamp, StartingValue){

set.seed(Seed)

if(DesiredSkew >= 0){

y<-StartingValue

d<-1

while(d){

sim <- scale(rbeta(NumOfSamp, y, 10))

if(skewness(sim) > DesiredSkew-0.0001 & skewness(sim) < DesiredSkew+0.0001) break

if(skewness(sim) > DesiredSkew) {y<-y+0.001}

if(skewness(sim) < DesiredSkew) {y<-y-0.001}

}

}

if(DesiredSkew < 0){

y<-StartingValue

d<-1

while(d){

sim <- scale(rbeta(NumOfSamp, 10, y))

if(skewness(sim) > DesiredSkew-0.0001 & skewness(sim) < DesiredSkew+0.0001) break

if(skewness(sim) > DesiredSkew) {y<-y-0.001}

if(skewness(sim) < DesiredSkew) {y<-y+0.001}

}

}

cat('StartingValue:',StartingValue,'\n')

cat('Skew:',skewness(sim),'\n')

cat('N:',length(sim),'\n')

cat('FinalValue=',y,'\n')

return(as.numeric(scale(sim)))

}

**Supplementary Text 2:** ‘SimQuest’ – Function to simulate questionnaire-type variables.

SimQuest<-function(DesiredSkew, NumOfResponse, Seed=10101, NumOfSamp, StartingValue){

set.seed(Seed)

Breaker<-function(x,n){

a<-NULL

for(y in seq(1:(n-1))){

a[y]<-(abs((max(x)-min(x)))/n)*y

}

return(c(min(x), min(x)+a, max(x)))

}

TieCreator<-function(x,y){

n<-length(y)

binned<-.bincode(round(x,2), round(y,2), right = TRUE, include.lowest = T)

return(binned)

}

if(DesiredSkew>=0){

y<-StartingValue

d<-1

while(d){

sim <- scale(rbeta(NumOfSamp, y, 10))

sim_breaks<-Breaker(sim,NumOfResponse)

tied_sim<-TieCreator(sim,sim_breaks)

if(skewness(tied_sim) > DesiredSkew-0.0001 & skewness(tied_sim) < DesiredSkew+0.0001) break

if(skewness(tied_sim) > DesiredSkew) {y<-y+0.001}

if(skewness(tied_sim) < DesiredSkew) {y<-y-0.001}

}

}

if(DesiredSkew<0){

y<-StartingValue

d<-1

while(d){

sim <- scale(rbeta(NumOfSamp, 10, y))

sim_breaks<-Breaker(sim,NumOfResponse)

tied_sim<-TieCreator(sim,sim_breaks)

if(skewness(tied_sim) > DesiredSkew-0.0001 & skewness(tied_sim) < DesiredSkew+0.0001) break

if(skewness(tied_sim) > DesiredSkew) {y<-y-0.001}

if(skewness(tied_sim) < DesiredSkew) {y<-y+0.001}

}

}

skewness(tied_sim)-DesiredSkew

cat('Number of available responses:',length(unique(tied_sim)),'\n')

cat('Skew:',skewness(tied_sim),'\n')

cat('N:',length(tied_sim),'\n')

cat('FinalValue=',y,'\n')

return(as.numeric(scale(tied_sim)))

}

**Supplementary Text 3:** ‘SimContNorm’ – Function to simulate continuous variables with skew and kurtosis equal to zero.

SimContNorm<-function(NumOfSamp, Seed=10101, StartingValue=10){

set.seed(Seed)

d<-1

while(d){

sim <- scale(rnorm(NumOfSamp))

if(skewness(sim) > -0.0001 & skewness(sim) < 0.0001 & kurtosis(sim) < 0.01 & kurtosis(sim) > -0.01) break

cat('Skew:',skewness(sim),'\n')

cat('Kurtosis:',skewness(sim),'\n')

}

cat('Skew:',skewness(sim),'\n')

cat('Kurtosis:',skewness(sim),'\n')

cat('N:',length(sim),'\n')

return(as.numeric(scale(sim)))

}

**Supplementary Text 4:** ‘SimQuestNorm’ – Function to simulate questionnaire-type variables with skew and kurtosis equal to zero.

SimQuestNorm<-function(NumOfResponse, Seed=10101, NumOfSamp){

set.seed(Seed)

Breaker<-function(x,n){

a<-NULL

for(y in seq(1:(n-1))){

a[y]<-(abs((max(x)-min(x)))/n)*y

}

return(c(min(x), min(x)+a, max(x)))

}

TieCreator<-function(x,y){

n<-length(y)

binned<-.bincode(round(x,2), round(y,2), right = TRUE, include.lowest = T)

return(binned)

}

d<-1

while(d){

sim <- scale(rnorm(NumOfSamp))

sim_breaks<-Breaker(sim,NumOfResponse)

tied_sim<-TieCreator(sim,sim_breaks)

if(skewness(tied_sim) > -0.0001 & skewness(tied_sim) < 0.0001 & kurtosis(tied_sim) < 0.01 & kurtosis(tied_sim) > -0.01) break

}

cat('Number of available responses:',length(unique(tied_sim)),'\n')

cat('Skew:',skewness(tied_sim),'\n')

cat('Kurtosis:',kurtosis(tied_sim),'\n')

cat('N:',length(tied_sim),'\n')

return(as.numeric(scale(tied_sim)))

}

**Supplementary Text 5:** ‘CovarCreator’ – Function to create correlated covariates for continuous and questionnaire-type variables.

CovarCreator<-function(x,dis.cor,dir,start=1){

y<-start

while(TRUE){

cov<-jitter(x, factor = y, amount = NULL)

j<-cor(cov,x,use='complete.obs')

if(j < dis.cor-0.0001) {y<-y-1}

if(j > dis.cor+0.0001) {y<-y+1}

if(j <= dis.cor+0.0001 & j >= dis.cor-0.0001) break()

}

print(y)

if(dir == 'neg') {cov<--cov}

j<-cor(cov,x,use='complete.obs')

print(j)

return(cov)

}

**Supplementary Text 6:** ‘rntransform_random’ – Function to perform rank-based INT whilst randomly splitting tied observations. Seed number should be set to enable perfect replication.

rntransform_random<-function (formula, data, family = gaussian, seed=NA)

{

if (is(try(formula, silent = TRUE), "try-error")) {

if (is(data, "gwaa.data"))

data1 <- phdata(data)

else if (is(data, "data.frame"))

data1 <- data

else stop("'data' must have 'gwaa.data' or 'data.frame' class")

formula <- data1[[as(match.call()[["formula"]], "character")]]

}

var <- ztransform(formula, data, family)

set.seed(seed)

out <- rank(var, ties.method='random') - 0.5

out[is.na(var)] <- NA

mP <- 0.5/max(out, na.rm = T)

out <- out/(max(out, na.rm = T) + 0.5)

out <- qnorm(out)

out

}

**Supplementary Table 1:** Difference in covariate correlation with the dependent variable before and after rank-based INT when splitting tied observations randomly. This is based on simulated data.

| **Original Covariate Correlation** | **Mean covariate correlation after normalization** | **Covariate correlation % difference after normalization** |
| --- | --- | --- |
| 0.5 | 0.476 | 4.88% |
| 0.25 | 0.239 | 4.61% |
| 0.12 | 0.114 | 5.07% |
| 0.06 | 0.057 | 5.04% |
| 0.03 | 0.029 | 7.27% |
| 0.01 | 0.010 | 21.30% |

**Supplementary Table 2:** Outcome of rank-based INT after regressing effect of a continuous covariate (age) from real questionnaire data.

| **Phenotype** | **Range** | **Original skew** | **Pearson correlation between phenotype and covariate** | **Pearson correlation between phenotype residuals and covariate** | **Pearson correlation between normalized phenotype residuals and covariate** | **Final skew** |
| --- | --- | --- | --- | --- | --- | --- |
| Paranoia | 5 | 1.357 | 0.055 | -1.55E-15 | -0.275 | 8.91E-06 |
| Paranoia | 10 | 1.195 | 0.043 | -1.89E-15 | -0.140 | 5.40E-06 |
| Paranoia | 20 | 1.095 | 0.030 | -1.59E-15 | -0.079 | 1.59E-05 |
| Paranoia | 40 | 1.296 | 0.022 | -1.42E-15 | -0.045 | 1.47E-05 |
|  |  |  |  |  |  |  |
| Anhedonia | 5 | 1.868 | -5.73E-03 | 5.81E-16 | 0.462 | 8.91E-06 |
| Anhedonia | 10 | 0.858 | -0.025 | 1.07E-15 | 0.172 | 5.40E-06 |
| Anhedonia | 20 | 0.651 | -0.020 | 1.06E-15 | 0.081 | 1.59E-05 |
| Anhedonia | 40 | 0.537 | -0.013 | 7.74E-16 | 0.037 | 1.47E-05 |

**Supplementary Table 3:** Outcome of regressing effect of a continuous covariate (age) from real questionnaire data on Spearman correlation.

| **Phenotype** | **Range** | **Original skew** | **Spearman correlation between phenotype and covariate** | **Spearman correlation between phenotype residuals and covariate** |
| --- | --- | --- | --- | --- |
| Paranoia | 5 | 1.357 | 0.063 | -0.266 |
| Paranoia | 10 | 1.195 | 0.049 | -0.123 |
| Paranoia | 20 | 1.095 | 0.031 | -0.061 |
| Paranoia | 40 | 1.296 | 0.027 | -0.025 |
|  |  |  |  |  |
| Anhedonia | 5 | 1.868 | -1.01E-03 | 0.436 |
| Anhedonia | 10 | 0.858 | -0.027 | 0.146 |
| Anhedonia | 20 | 0.651 | -0.027 | 0.062 |
| Anhedonia | 40 | 0.537 | -0.016 | 0.031 |

**Supplementary Table 4:** Outcome of rank-based INT after regressing effect of a dichotomous covariate (sex) from real questionnaire data.

| **Phenotype** | **Range** | **Original skew** | **Pearson correlation between phenotype and covariate** | **Pearson correlation between phenotype residuals and covariate** | **Pearson correlation between normalized phenotype residuals and covariate** | **Final skew** |
| --- | --- | --- | --- | --- | --- | --- |
| Paranoia | 5 | 1.357 | 0.018 | -1.00E-16 | -0.264 | 0.624 |
| Paranoia | 10 | 1.195 | -0.026 | -8.74E-17 | 0.125 | 0.209 |
| Paranoia | 20 | 1.095 | -0.022 | -2.93E-16 | 0.065 | 0.097 |
| Paranoia | 40 | 1.296 | -0.059 | -5.60E-16 | -7.96E-03 | 0.077 |
|  |  |  |  |  |  |  |
| Anhedonia | 5 | 1.868 | 0.177 | 4.78E-16 | -0.212 | 0.624 |
| Anhedonia | 10 | 0.858 | 0.127 | 4.07E-16 | -0.040 | 0.209 |
| Anhedonia | 20 | 0.651 | 0.135 | 4.09E-16 | 0.049 | 0.097 |
| Anhedonia | 40 | 0.537 | 0.205 | -1.77E-16 | -5.51E-03 | 0.077 |

**Supplementary Table 5:** Outcome of regressing effect of a dichotomous covariate (sex) from real questionnaire data on Spearman correlation.

| **Phenotype** | **Range** | **Original skew** | **Spearman correlation between phenotype and covariate** | **Spearman correlation between phenotype residuals and covariate** |
| --- | --- | --- | --- | --- |
| Paranoia | 5 | 1.357 | 0.018 | -0.269 |
| Paranoia | 10 | 1.195 | -0.031 | 0.118 |
| Paranoia | 20 | 1.095 | -0.026 | 0.054 |
| Paranoia | 40 | 1.296 | -0.066 | -0.020 |
|  |  |  |  |  |
| Anhedonia | 5 | 1.868 | 0.200 | -0.187 |
| Anhedonia | 10 | 0.858 | 0.124 | -0.025 |
| Anhedonia | 20 | 0.651 | 0.140 | 0.065 |
| Anhedonia | 40 | 0.537 | 0.211 | 9.05E-03 |

**Supplementary Table 6:** Effect of rank-based INT (randomly ranking tied observations) on the relationship between real questionnaire variable and continuous covariate (age). This table also shows to what extent regressing covariate effects reintroduces skew.

| Phenotype | Range | Original Skew of Dependent Variable | Original Correlation between Dependent Variable and Age | Correlation between Normalized Dependant Variable and Age | Correlation between Residuals of Normalized Dependant Variable and Age | Skew of Residuals of Normalized Dependant |
| --- | --- | --- | --- | --- | --- | --- |
| Paranoia | 5 | 1.357 | 0.055 | 0.054 | -2.52E-15 | -3.87E-03 |
| Paranoia | 10 | 1.195 | 0.043 | 0.043 | -2.99E-15 | -2.56E-03 |
| Paranoia | 20 | 1.095 | 0.030 | 0.029 | -1.96E-15 | -1.67E-03 |
| Paranoia | 40 | 1.296 | 0.022 | 0.023 | -1.66E-15 | -3.67E-04 |
|  |  |  |  |  |  |  |
| Anhedonia | 5 | 1.868 | -0.006 | -0.013 | 1.01E-15 | 5.34E-04 |
| Anhedonia | 10 | 0.858 | -0.025 | -0.028 | 1.56E-15 | 3.27E-04 |
| Anhedonia | 20 | 0.651 | -0.020 | -0.024 | 1.24E-15 | 1.67E-03 |
| Anhedonia | 40 | 0.537 | -0.013 | -0.014 | 8.05E-16 | 1.02E-04 |

**Supplementary Table 7:** Effect of rank-based INT (randomly ranking tied observations) on the relationship between real questionnaire variable and binary covariate (sex). This table also shows to what extent regressing covariate effects reintroduces skew.

| Phenotype | Range | Original Skew of Dependent Variable | Original Correlation between Dependent Variable and Sex | Correlation between Normalized Dependant Variable and Sex | Correlation between Residuals of Normalized Dependant Variable and Sex | Skew of Residuals of Normalized Dependant |
| --- | --- | --- | --- | --- | --- | --- |
| Paranoia | 5 | 1.357 | 0.018 | 0.012 | 7.14E-16 | -2.81E-04 |
| Paranoia | 10 | 1.195 | -0.026 | -0.033 | 5.17E-16 | 1.54E-03 |
| Paranoia | 20 | 1.095 | -0.022 | -0.026 | 5.96E-17 | 1.36E-03 |
| Paranoia | 40 | 1.296 | -0.059 | -0.065 | -4.01E-16 | 1.18E-03 |
|  |  |  |  |  |  |  |
| Anhedonia | 5 | 1.868 | 0.177 | 0.175 | 4.80E-16 | -0.017 |
| Anhedonia | 10 | 0.858 | 0.127 | 0.127 | 5.92E-16 | -0.010 |
| Anhedonia | 20 | 0.651 | 0.135 | 0.136 | 4.94E-16 | -1.35E-03 |
| Anhedonia | 40 | 0.537 | 0.205 | 0.206 | -7.20E-17 | -3.32E-03 |

**Supplementary Table 8:** Effect of rank-based INT of real questionnaire variable when randomly ranking tied observations. Shows Pearson correlation between dependent variable before after normalization.

| Phenotype | Range | Original Skew | Correlation after rank-based INT |
| --- | --- | --- | --- |
| Paranoia | 5 | 1.357 | 0.889 |
| Paranoia | 10 | 1.195 | 0.939 |
| Paranoia | 20 | 1.095 | 0.957 |
| Paranoia | 40 | 1.296 | 0.949 |
|  |  |  |  |
| Anhedonia | 5 | 1.868 | 0.833 |
| Anhedonia | 10 | 0.858 | 0.958 |
| Anhedonia | 20 | 0.651 | 0.980 |
| Anhedonia | 40 | 0.537 | 0.990 |

Supplementary Figure 1: Effect of regressing out covariate effects from questionnaire-type data with a range of 5 before rank-based non-parametric analyses. X-axis shows the skew of the original phenotypic data. Y-axis shows the Spearman rank-based correlation between residuals and covariates. Colors indicate the original Pearson correlation between the questionnaire-type data and covariate.

Supplementary Figure 2: Effect of regressing out covariate effects from questionnaire-type data with a range of 10 before rank-based non-parametric analyses. X-axis shows the skew of the original phenotypic data. Y-axis shows the Spearman rank-based correlation between residuals and covariates. Colors indicate the original Pearson correlation between the questionnaire-type data and covariate.

Supplementary Figure 3: Effect of regressing out covariate effects from questionnaire-type data with a range of 20 before rank-based non-parametric analyses. X-axis shows the skew of the original phenotypic data. Y-axis shows the Spearman rank-based correlation between residuals and covariates. Colors indicate the original Pearson correlation between the questionnaire-type data and covariate.

Supplementary Figure 4: Effect of regressing out covariate effects from questionnaire-type data with a range of 40 before rank-based non-parametric analyses. X-axis shows the skew of the original phenotypic data. Y-axis shows the Spearman rank-based correlation between residuals and covariates. Colors indicate the original Pearson correlation between the questionnaire-type data and covariate.

Supplementary Figure 5: Effect of regressing out covariate effects from questionnaire-type data with a range of 80 before rank-based non-parametric analyses. X-axis shows the skew of the original phenotypic data. Y-axis shows the Spearman rank-based correlation between residuals and covariates. Colors indicate the original Pearson correlation between the questionnaire-type data and covariate.

Supplementary Figure 6: Effect of regressing out covariate effects from questionnaire-type data with a range of 160 before rank-based non-parametric analyses. X-axis shows the skew of the original phenotypic data. Y-axis shows the Spearman rank-based correlation between residuals and covariates. Colors indicate the original Pearson correlation between the questionnaire-type data and covariate.

Supplementary Figure 7: Effect of regressing out covariate effects from continuous data before rank-based non-parametric analyses. X-axis shows the skew of the original phenotypic data. Y-axis shows the Spearman rank-based correlation between residuals and covariates. Colors indicate the original Pearson correlation between the continuous data and covariate.

Supplementary Figure 8: Effect of rank-based INT of questionnaire-type data residuals (after regressing out covariates) with range of 5. The relationship between the original skew of the raw questionnaire-type data (x-axis), the original correlation between the raw questionnaire-type data and covariate data (color coded), and the correlation between normalised questionnaire-type residuals (y-axis). This figure is based on simulated questionnaire-type data with a range of 0-5.

Supplementary Figure 9: Effect of rank-based INT of questionnaire-type data residuals (after regressing out covariates) with range of 10. The relationship between the original skew of the raw questionnaire-type data (x-axis), the original correlation between the raw questionnaire-type data and covariate data (color coded), and the correlation between normalised questionnaire-type residuals (y-axis). This figure is based on simulated questionnaire-type data with a range of 0-10.

Supplementary Figure 10: Effect of rank-based INT of questionnaire-type data residuals (after regressing out covariates) with range of 20. The relationship between the original skew of the raw questionnaire-type data (x-axis), the original correlation between the raw questionnaire-type data and covariate data (color coded), and the correlation between normalised questionnaire-type residuals (y-axis). This figure is based on simulated questionnaire-type data with a range of 0-20.

Supplementary Figure 11: Effect of rank-based INT of questionnaire-type data residuals (after regressing out covariates) with range of 40. The relationship between the original skew of the raw questionnaire-type data (x-axis), the original correlation between the raw questionnaire-type data and covariate data (color coded), and the correlation between normalised questionnaire-type residuals (y-axis). This figure is based on simulated questionnaire-type data with a range of 0-40.

Supplementary Figure 12: Effect of rank-based INT of questionnaire-type data residuals (after regressing out covariates) with range of 80. The relationship between the original skew of the raw questionnaire-type data (x-axis), the original correlation between the raw questionnaire-type data and covariate data (color coded), and the correlation between normalised questionnaire-type residuals (y-axis). This figure is based on simulated questionnaire-type data with a range of 0-80.

Supplementary Figure 13: Effect of rank-based INT of questionnaire-type data residuals (after regressing out covariates) with range of 160. The relationship between the original skew of the raw questionnaire-type data (x-axis), the original correlation between the raw questionnaire-type data and covariate data (color coded), and the correlation between normalised questionnaire-type residuals (y-axis). This figure is based on simulated questionnaire-type data with a range of 0-160.

**Supplementary Figure 14:** Effect of rank-based INT of continuous data residuals (after regressing out covariates). The relationship between the original skew of the raw continuous data (x-axis), the original correlation between the raw continuous data and covariate data (color coded), and the correlation between normalised residuals (y-axis). This figure is based on simulated continuous data.

**Supplementary Figure 15:** Effect of rank-based INT when kurtosis and skew are equal to zero.

Supplementary Figure 16: Relationship between proportion of ties and magnitude of original covariate correlation. The number of response bins (x-axis) is a measure of the proportion of tied observations. As the number of available increases, the proportion of tied observations decreases. The y-axis is the absolute correlation between normalised residual and the covariate, and therefore indicates the degree to which normalization reintroduces the covariate correlation with residuals. Color indicates the original correlation between the covariate and simulated variables. This figure is based on simulated variables with a skew of 1.

Supplementary Figure 17: The relationship between the number of available responses in questionnaire-type data (x-axis) and the absolute Spearman rank-based correlation between normalized residuals and covariate (y-axis) for different values of the skew. Within this figure, the Pearson correlation between the raw questionnaire-type data and covariate data is at 0.06.

Supplementary Figure 18: Relationship between proportion of ties and magnitude of original covariate correlation. The number of response bins (x-axis) is a measure of the proportion of tied observations. The y-axis is the absolute Spearman rank-based correlation between residuals and covariates. Color indicates the original Pearson correlation between the covariates and raw simulated variables. This figure is based on simulated variables with a skew of 1.

**Supplementary figure 19:** Difference in covariate correlation with dependent variable after rank-based INT. Original covariate correlation in this figure was 0.5.

**Supplementary figure 20:** Difference in covariate correlation with dependent variable after rank-based INT. Original covariate correlation in this figure was 0.25.

**Supplementary figure 21:** Difference in covariate correlation with dependent variable after rank-based INT. Original covariate correlation in this figure was 0.12.

**Supplementary figure 22:** Difference in covariate correlation with dependent variable after rank-based INT. Original covariate correlation in this figure was 0.06.

**Supplementary figure 23:** Difference in covariate correlation with dependent variable after rank-based INT. Original covariate correlation in this figure was 0.03.

**Supplementary figure 24:** Difference in covariate correlation with dependent variable after rank-based INT. Original covariate correlation in this figure was 0.01.

**Supplementary figure 25:** Correlation between the dependent variable before and after rank-based INT (randomly splitting tied observations).

**Supplementary figure 26:** Magnitude of skew reintroduced when regressing out covariate effects from normalized dependent variables. Proportion of ties did not affect this. This figure is based on simulated continuous variables.
